# Supplementary material for: Gut microbiota facilitate chronic spontaneous urticaria
Source: Nat Commun. 2024 Jan 2;15:112. doi: 10.1038/s41467-023-44373-x (PMC10762022; doi:10.1038/s41467-023-44373-x)

Supplementary Information

Gut Microbiota Facilitate Chronic Spontaneous Urticaria

Authors:

Lei Zhu[1,2,3], Xingxing Jian[4], Bingjing Zhou[1,2,3], Runqiu Liu[5], Melba Muñoz[6,7], Wan Sun[8], Lu Xie[4], Xiang Chen[1,2,3#], Cong Peng[1,2,3#], Marcus Maurer[6.7#], Jie Li[1,2,3#]

Affiliations:

[1]Department of Dermatology, Xiangya Hospital, Central South University, Changsha, Hunan, China.

[2] Hunan Key Laboratory of Skin Cancer and Psoriasis, Furong Labratory, Changsha, Hunan, China.

[3]National Clinical Research Center for Geriatric Disorders, Xiangya Hospital, Central South University, Changsha, Hunan, China.

[4]Bioinformatics Center, Xiangya Hospital, Central South University, Changsha, Hunan, China.

[5]Department of Dermatology, the First people's Hospital of Yancheng, Yancheng Clinical College of Xuzhou Medical University, Yancheng, Jiangsu, China.

[6]Institute of Allergology, Charité-Universitätsmedizin Berlin, corporate member of Freie Universität Berlin and Humboldt-Universität zu Berlin, Berlin, Germany.

[7]Fraunhofer Institute for Translational Medicine and Pharmacology ITMP, Allergology and Immunology, Berlin, Germany.

[8]BGI, Complex building, Beishan Industrial Zone, Yantian District, Shenzhen, China.

Corresponding Authors:

xylijie@csu.edu.cn (J. Li) marcus.maurer@charite.de (M. Maurer)

[#] These authors contributed equally to this work.

Supplementary Methods

Study participants and conduct

We obtained detailed data on the medical history, comorbidities, and clinical manifestation of all the patients. We also performed laboratory tests such as routine blood analyses, autoantibodies (e.g., anti-thyroid autoantibodies, SLE-related antibodies), sedimentation, CRP, and thyroid hormone levels.

***Tlr4*$^{-/-}$ mouse genotyping**

Mouse tail tissues (~0.5 g) were clipped with sterile scissors, and genomic DNA was extracted using the Mouse Tail Direct PCR Kit (Selleck, China). Extracted DNA was subjected to PCR using primers(Forward:AGGGAGATGTGTGTGTGAAGAAGCCT; Reverse:TTCCAGCTATGGCCCAGATGAACT). The 416 bp band represents the pure heterozygote and no band at this position represents wild type.

Western Blot

BMMC cell samples were lysed with RIPA buffer containing 1 mM protease inhibitor (Beyotime Technology, China) at 4°C for 30 min, and centrifuged at 14,000 rpm for 10 min. The supernatant was collected and the protein concentration was determined using the BCA Protein Concentration Assay Kit (Beyotime Technology, China). Protein extracts were mixed with the uploading buffer and heated at 95°C for 10 min to denature the proteins. Denatured proteins were separated by 10% SDS-PAGE gel electrophoresis and transferred to a 0.45um PVDF membrane(Millipore, USA). The membrane was closed with 5% BSA for 1 h at room temperature and incubated with primary antibody for 16 h at 4°C, followed by incubation with horseradish peroxidase-coupled secondary antibody for 1 h at room temperature. The bands were visualized using a chemiluminescence kit (NCM Biotech, China) and a chemiluminescence imaging system, ChemiDoc MP (BIORAD, USA). Molecular Weight of glycosylated TLR4: 95/120 kDa, Molecular Weight of Actin: 43 kDa.

Antibodies and dilution ratios used were as follows: TLR4 antibody, (Santa Cruz, Cat#: sc-293072; Clone:25, dilution ratio: 1:500); actin antibody, (Santa Cruz, Cat#: sc-8432; Clone:C-2, dilution ratio: 1:1000).

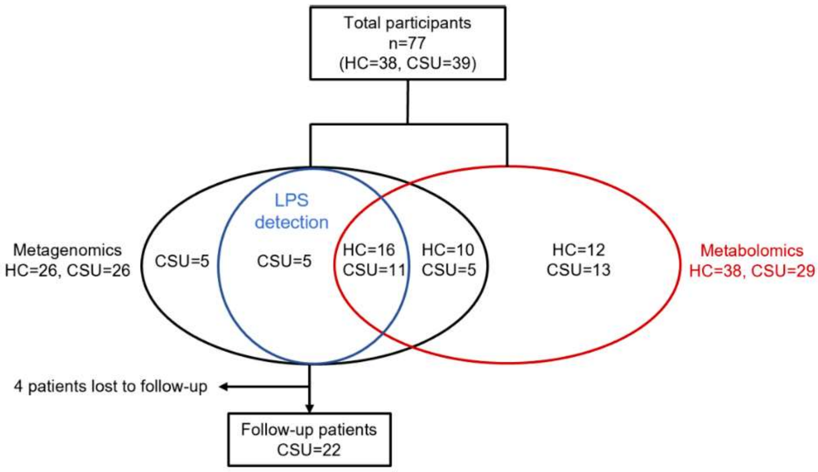

Supplementary Fig. 1 Details of participants' screening and research process. A total of 77 participants were enrolled, including 38 HC and 39 CSU patients. Of them, 26 HC and 26 CSU patients received metagenomic sequencing in fecal samples; 38 HC and 29 CSU patients received targeted metabolomics in plasma; 26 HC and 16 CSU patients receiving both metagenomic sequencing in fecal samples and targeted metabolomics in plasma. Of the HC and CSU patients that received metagenomic sequencing in fecal samples, LPS in plasma were examined in 16 HC and 16 CSU patients. 16 HC and 11 CSU patients received metagenomic sequencing in fecal samples, targeted metabolomics in plasma, and LPS examination in plasma. 26 CSU patients who had undergone fecal metagenomic sequencing were followed up, and 22 patients completed follow-up for remission and relapse. HC: healthy controls; CSU: chronic spontaneous urticaria.

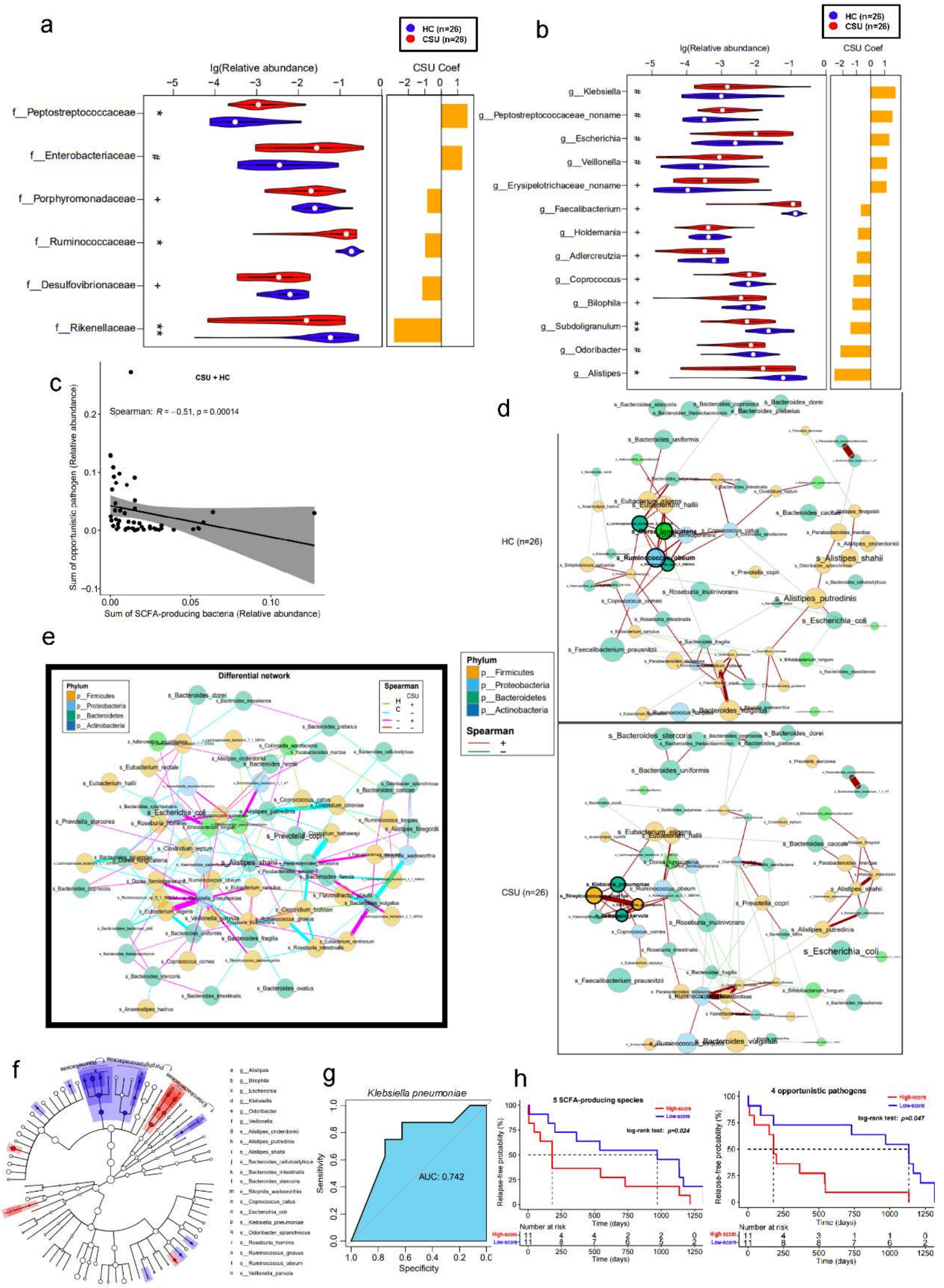

Supplementary Fig.2 Analysis between CSU patients and HC based on metagenomic sequencing and the association analysis of CSU recurrence with clinical features. Differential families **a** and genus **b** identified by MaAsLin2 with BH correction between HC and CSU group (*P* values were provided in Source Data File). #*P*<0.2, +*P*<0.1, **P*<0.05, ***P*<0.01. **c** Two-tailed spearman's correlation between the sum of SCFA-producing bacteria and the sum of opportunistic pathogens in all samples (HC=26,

CSU=26, *P*=0.00014). The line represents centre of overall linear fit, with grey areas standing for the standard error of 95% confidence interval. **d** The Spearman's correlation networks based on the core species in HC (upper panel) and CSU (lower panel), respectively (r>0.5, *P*<0.05), which was constructed by R package NetCoMi and "eigenvector" was used to identify hubs to each group. Core species were those that were detected in at least 50% of samples in this cohort. Each node represents a core species, and nodes of the same color are derived from the same phylum. An edge between nodes denotes the different association in CSU and HC, and the color and size of edges represent the different association types and association intensity, respectively. "+" indicates a positive correlation, and "-" indicates a negative correlation. **e** The differential Spearman's correlations network between HC (n=26) and CSU (n=26).**f** Cladogram of significant differences between the two groups from the family level to the species level. HC(n=26) vs CSU(n=26). The blue dot indicates that the taxon presents a higher relative abundance in HC(n=26), and the red dot indicates that the taxon presents a higher relative abundance in CSU. **g** Receiver operating characteristic curves of *Klebsiella pneumoniae* for correctly distinguishing HC(n=26) and CSU(n=26, AUC=0.742). The shape and whiskers of violin plot respectively represent density distribution and overall range of the data in (**a, b**). AUC: area under curve. **h** Relapse-free analysis based on the 5 SCFA-producing bacteria (Left: n=22, *P*=0.024) or 4 opportunistic pathogens (Right: n=22, *P*=0.047) was determined by logrank test. ***P*<0.01. Source data are provided as a Source Data file.

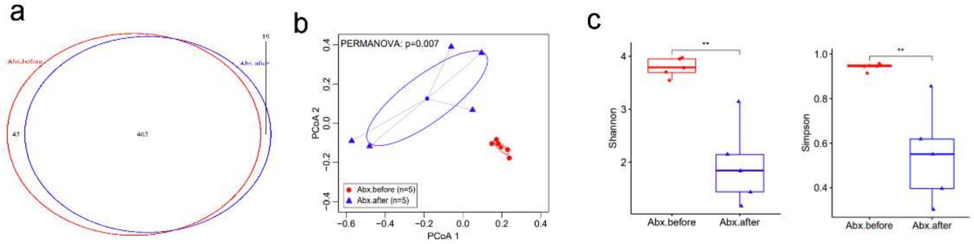

Supplementary Fig.3 Alteration of gut microbiota in mice before and after antibiotic treatment by 16S rDNA sequencing. a Number of OTUs shared and unique between Abx-before and Abx-after. **b** Comparison of β-diversity between Abx-before and Abx-after (n=5, P=0.007). Significance was determined by PERMANOVA. **c** Left panel: comparison of Shannon index between Abx-before and Abx-after (n=5, P=0.0079); Right panel: comparison of Simpson index between Abx-before and Abx-after (n=5, P=0.0079). Box plots indicate median (middle line), 25th, 75th percentile (box) and 5th and 95th percentile (whiskers) as well as outliers (single points). Significance was determined by two-tailed Wilcoxon test. **P<0.01. Abx-before: mice before antibiotic treatment. Abx-after: mice after antibiotic treatment. Source data are provided as a Source Data file.

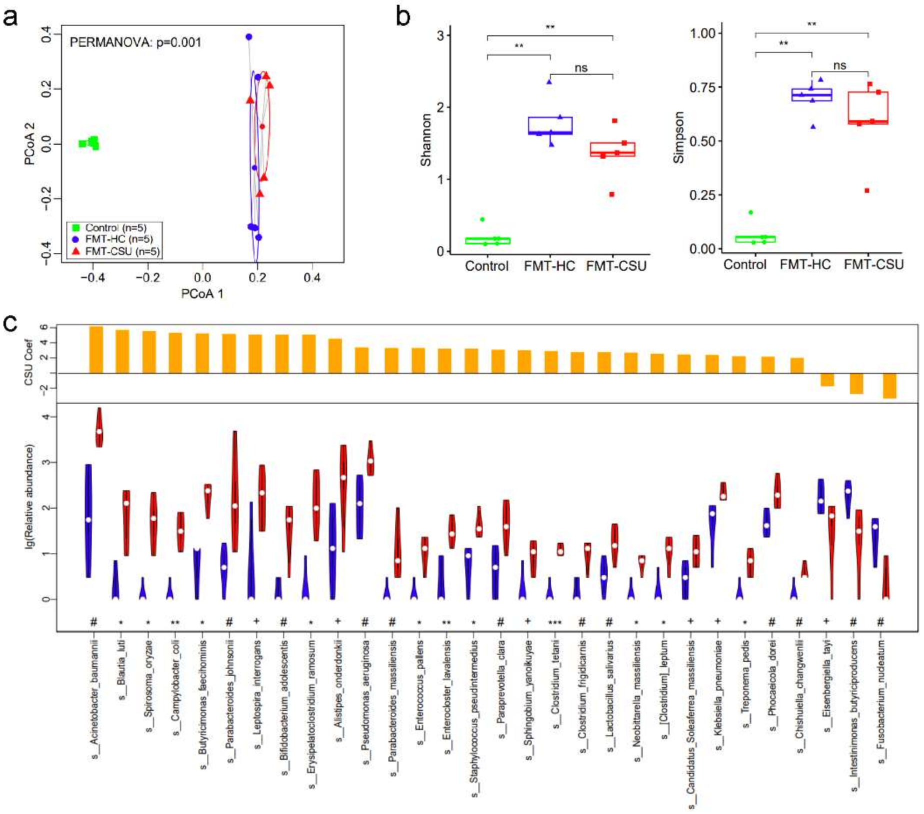

Supplementary Fig. 4 Comparison of gut microbiota between fecal microbial transplantation (FMT)-HC mice and FMT-CSU mice after FMT by metagenomic sequencing. a Comparison of β-diversity between Control, FMT-HC and FMT-CSU. (n=5). **b** Left panel: comparison of Shannon indexes between Control, FMT-HC and FMT-CSU (n=5, Control vs FMT-HC, *P*=0.0079, Control vs FMT-CSU, P=0.0079); Right panel: comparison of Simpson indexes between Control, FMT-HC and FMT-CSU(n=5, Control vs FMT-HC, *P*=0.0079, Control vs FMT-CSU, *P*=0.0079). Significance was determined by Wilcoxon test. Box plots indicate median (middle line), 25th, 75th percentile (box) and 5th and 95th percentile (whiskers) as well as outliers (single points).**c** Different species between FMT-HC and FMT-CSU(n=5, *P* values were provided in Source Data File). #*P*<0.2, +*P*<0.1, **P*<0.05, ***P*<0.01. The shape and whiskers of violin plot respectively represent density distribution and overall range of the data. Significance was determined by PERMANOVA(**a**) or two-tailed Wilcoxon test(**b, c**). Control: gavage with solvents; FMT-HC: FMT from healthy control; FMT-CSU: FMT from CSU patients. ns: no significant. Source data are provided as a Source Data file.

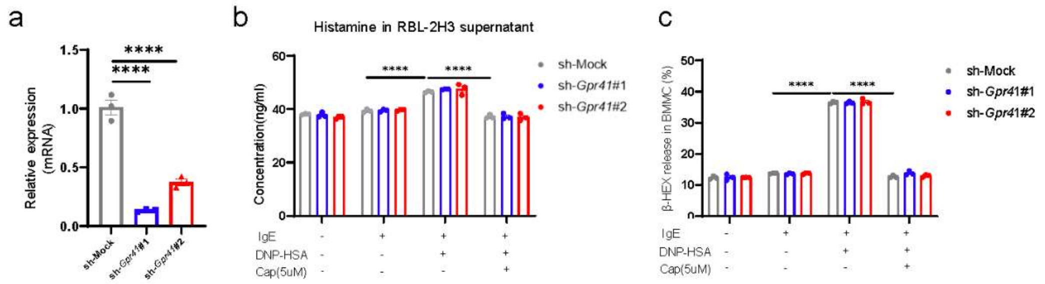

Supplementary Fig. 5 Knockdown of *Gpr41* has no effect on degranulation of BMMCs and histamine release from RBL-2H3 after caproate administration. a *Gpr41* knockdown efficiency validation. mRNA expression of *Gpr41* were analyzed through RT-PCR (n=3/group, sh-Mock vs sh-*Gpr41*#1, *P*<0.0001; sh-Mock vs sh-*Gpr41*#2, *P*<0.0001). **b** Knockdown of *Gpr41* failed to block the effect of caproate(5uM) on histamine release (n=3/group, IgE$^+$/DNP-HSA$^-$/Cap(5uM)$^-$ vs IgE$^+$/DNP-HSA$^+$/Cap(5uM)$^-$, *P*<0.0001; IgE$^+$/DNP-HSA$^+$/Cap(5uM)$^-$ vs IgE$^+$/DNP-HSA$^+$/Cap(5uM)$^+$, *P*<0.0001)and **c** β-hexosaminidase release from MCs(n=3/group, IgE$^+$/DNP-HSA$^-$/Cap(5uM)$^-$ vs IgE$^+$/DNP-HSA$^+$/Cap(5uM)$^-$, *P*<0.0001; IgE$^+$/DNP-HSA$^+$/Cap(5uM)$^-$ vs IgE$^+$/DNP-HSA$^+$/Cap(5uM)$^+$, *P*<0.0001). Significance was determined by one-way ANOVA with Tukey's multiple comparisons test. *$P < 0.05$, **$P < 0.01$, ***$P < 0.001$, ****$P < 0.0001$. The data are presented as mean±SEM of three independent experiments. BMMCs: bone marrow-derived mast cells. Source data are provided as a Source Data file.

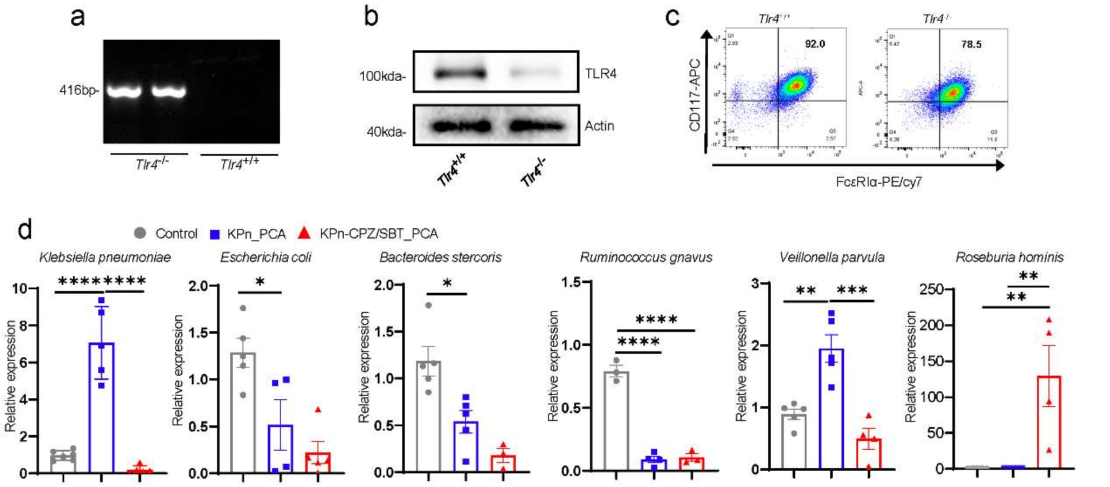

Supplementary Fig. 6 Knockdown of *Tlr4 in vivo* and in *vitro*, BMMCs differentiation, changes in relative abundance of representative pathogenic bacteria after KPn transplantation and antibiotic treatment. a Identification of representative genotypes of *Tlr4*$^{-/-}$ and *Tlr4*$^{+/+}$ mice. **b** Validation of TLR4 knockdown efficiency on mature BMMCs. **c** *Tlr4*$^{+/+}$ and *Tlr4*$^{-/-}$ BMMCs differentiation rate detected by flow cytometry. **d** mRNA expression of *Klebsiella pneumoniae*(n=5/group, *P*<0.0001), *Escherichia coli*(Control, n=5, KPn_PCA, n=4, KPn-CPZ/SBT_PCA, n=5. Control vs KPn_PCA, *P*=0.0302), *Bacteroides stercoris*(Control, n=5, KPn_PCA, n=5, KPn-CPZ/SBT_PCA, n=3. Control vs KPn_PCA, *P*=0.0136), *Ruminococcus gnavus*(Control, n=3, KPn_PCA, n=4, KPn-CPZ/SBT_PCA, n=3. Control vs KPn_PCA and Control vs KPn-CPZ/SBT_PCA, both *P*<0.0001), *Veillonella_parvula*(Control, n=5, KPn_PCA, n=5, KPn-CPZ/SBT_PCA, n=4. Control vs KPn_PCA, *P*=0.0022; KPn_PCA vs KPn-CPZ/SBT_PCA, *P*=0.0003), and *Roseburia hominis*(Control, n=5, KPn_PCA, n=5, KPn-CPZ/SBT_PCA, n=4. Control vs KPn-CPZ/SBT_PCA, *P*=0.0032; KPn_PCA vs KPn-CPZ/SBT_PCA, *P*=0.0032) were analyzed through RT-PCR in mice feces. Significance was determined by ordinary one-way ANOVA with Tukey's multiple comparisons test. **P* < 0.05, ***P* < 0.01, ****P* < 0.001, *****P* < 0.0001. The data are presented as mean±SEM of three independent experiments. Source data are provided as a Source Data file.

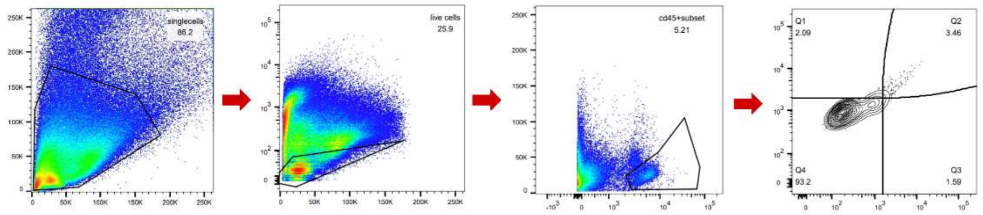

Supplementary Fig. 7 Representative gating strategies for mouse skin tissue flow cytometry analysis. The first gate circled single cells, the second gate circled live cells, the third gate circled CD45⁺ cells, and the fourth gate circled CD117⁺FcεRI⁺ cells. Source data are provided as a Source Data file.

Supplementary Table 1 Basic characteristics of participants for metagenomic sequencing

Clinical characteristics	HC (n=26)	CSU (n=26)	HC vs. CSU statistical significance
Gender (male/female)	10/16	10/16	ns§
Age (years)†	39.0±6.2	36.1±9.2	ns§
Waist circumference(cm)†	79.1±7.6	82.7±10.1	ns§
Body Mass Index (BMI)†	23.1±2.7	23.8±3.7	ns§
7-day urticaria activity score (UAS7) †	—	22.3±12.5	—
Duration of the disease (months)‡	—	48(12-120)	—
Dermatology life quality index (DLQI)‡	—	4(2.8-9.5)	—

†: mean ± standard deviation; ‡: median (25% percentile to 75% percentile); §: no significance. HC: healthy control, CSU: chronic spontaneous urticaria. Normality distribution was tested.

Supplementary Table 2 The bacteria alteration between HC and CSU

Species	Opportunistic pathogens	SCFA producers	Harmful effect on intestinal permeability	Reference
Odoribacter splanchnicus	No	Yes	No	Ref: [2,3]
Ruminococcus obeum	No	Yes	No	Ref: [4-6]
Alistipes putredinis	Yes	Yes	No	Ref: [7,8]
Alistipes shahii	Yes	Yes	No	Ref: [9,10]
Alistipes onderdonkii	Yes	Yes	No	Ref: [8,11]
Bacteroides cellulosilyticus	Yes	Yes	No	Ref: [12,13]
Coprococcus catus	No	Yes	No	Ref: [5,14,15]
Roseburia hominis	No	Yes	No	Ref: [16,17]
Escherichia coli	Yes	No	Yes	Ref: [18,19]
Klebsiella pneumoniae	Yes	No	Yes	Ref: [19,20]
Bacteroides stercoris	Yes	Yes	No	Ref: [21,22]
Ruminococcus gnavus	Yes	No	Yes	Ref: [23,24]
Bilophila wadsworthia	Yes	No	Yes	Ref:[1,25]
Veillonella parvula	Yes	No	Yes	Ref:[26,27]
Bacteroides intestinalis	Yes	Yes	No	Ref:[28]

Supplementary Table 3 List of primers for markers in mouse skin tissue and bacteria in gut flora

name	Primer	Sequence (5′-3′)
β-actin	Forward primer	CTACCTCATGAAGATCCTGACC
	Reverse primer	CACAGCTTCTCTTTGATGTCAC
Il13	Forward primer	CAACGGCAGCATGGTAT
	Reverse primer	GCAATATCCTCTGGGTCCT
Tnfα	Forward primer	GAAACACAAGATGCTGGGA
	Reverse primer	TTGCAGAACTCAGGAATGG
Il4	Forward primer	TACCAGGAGCCATATCCACGGATG
	Reverse primer	TGTGGTGTTCTTCGTTGCTGTGAG
Il10	Forward primer	GCCCTTTGCTATGGTGTC
	Reverse primer	TCTCCCTGGTTTCTCTTCC
Zo1	Forward primer	TGGAATTGCAATCTCTGGTG
	Reverse primer	CTGGCCCTCCTTTTAACACA
Occludin	Forward primer	CCTATCTTGGGAGCCTGGACAT
	Reverse primer	GATTGGGTTTGAATTCATCAGGTC
Cgn	Forward primer	ACAAAAGACCCTCCTTATGGCTT
	Reverse primer	AGAGGCAAACCATCCCCAT
Tjp2	Forward primer	ATGGGAGCAGTACACCGTGA
	Reverse primer	TGACCACCCTGTCATTTTCTTG
Total Bacteria	Forward primer	GTGSTGCAYYGGYTGTCGTCA
	Reverse primer	ACCGTCRTCCMCACCTTCCTC
Klebsiella pneumoniae	Forward primer	GCGGACGGGTGAGTAATGTC
	Reverse primer	AGCCGTTACCCCACCTACTA
Bacteroides stercoris	Forward primer	AAAGCTTGCTTTGATGGATG
	Reverse primer	ACATACAAAAAGCCACACGTC
Veillonella parvula	Forward primer	TCCTCTTCTTCGGAAGCCAGA
	Reverse primer	AGAGAGTGTTTCTCGGGTTTGC
Roseburia hominis	Forward primer	GCTTCCGTGCGTGCTTGT
	Reverse primer	CCGTACTTCACGGCAAGCTT
Ruminococcus gnavus	Forward primer	TGGCGGCGTGCTTAACA
	Reverse primer	TCCGAAGAAATCCGTCAAGGT
Escherichia coli	Forward primer	TGTTACGTCCTGTAGAAAGCCC
	Reverse primer	AAAACTGCCTGGCACAGCAATT

Supplementary References

1 Natividad, J. M. *et al.* Bilophila wadsworthia aggravates high fat diet induced metabolic dysfunctions in mice. *Nat Commun* **9**, 2802 (2018).

2 Lima, S. F. *et al.* Transferable Immunoglobulin A-Coated Odoribacter splanchnicus in Responders to Fecal Microbiota Transplantation for Ulcerative Colitis Limits Colonic Inflammation. *Gastroenterology* **162**, 166-178 (2022).

3 Hiippala, K. *et al.* Novel Odoribacter splanchnicus Strain and Its Outer Membrane Vesicles Exert Immunoregulatory Effects in vitro. *Front Microbiol* **11**, 575455 (2020).

4 Zou, T. *et al.* Polysaccharide-rich fractions from Enteromorpha prolifera improve hepatic steatosis and gut barrier integrity in high-fat diet-induced obese mice linking to modulation of gut microbiota. *Biomed Pharmacother* **157**, 114034 (2023).

5 Liu, Y. *et al.* Alteration of Gut Microbiota Relates to Metabolic Disorders in Primary Aldosteronism Patients. *Front Endocrinol (Lausanne)* **12**, 667951 (2021).

6 El Hage, R., Hernandez-Sanabria, E., Calatayud Arroyo, M., Props, R. & Van de Wiele, T. Propionate-Producing Consortium Restores Antibiotic-Induced Dysbiosis in a Dynamic in vitro Model of the Human Intestinal Microbial Ecosystem. *Front Microbiol* **10**, 1206 (2019).

7 Yin, L. *et al.* Poria cocos polysaccharides exert prebiotic function to attenuate the adverse effects and improve the therapeutic outcome of 5-FU in Apc(Min/+) mice. *Chin Med* **17**, 116 (2022).

8 Parker, B. J., Wearsch, P. A., Veloo, A. C. M. & Rodriguez-Palacios, A. The Genus Alistipes: Gut Bacteria With Emerging Implications to Inflammation, Cancer, and Mental Health. *Front Immunol* **11**, 906 (2020).

9 Ticinesi, A. *et al.* The Gut-Muscle Axis in Older Subjects with Low Muscle Mass and Performance: A Proof of Concept Study Exploring Fecal Microbiota Composition and Function with Shotgun Metagenomics Sequencing. *Int J Mol Sci* **21** (2020).

10 Li, Y. D., He, K. X. & Zhu, W. F. Correlation between invasive microbiota in margin-surrounding mucosa and anastomotic healing in patients with colorectal cancer. *World J Gastrointest Oncol* **11**, 717-728 (2019).

11 Cobo, F. *et al.* First description of abdominal infection due to Alistipes onderdonkii. *Anaerobe* **66**, 102283 (2020).

12 He, X. *et al.* In Vitro Colonic Fermentation Profiles and Microbial Responses of Cellulose Derivatives with Different Colloidal States. *J Agric Food Chem* **70**, 9509-9519 (2022).

13 Robert, C., Chassard, C., Lawson, P. A. & Bernalier-Donadille, A. Bacteroides cellulosilyticus sp. nov., a cellulolytic bacterium from the human gut microbial community. *Int J Syst Evol Microbiol* **57**, 1516-1520 (2007).

14 Sheridan, P. O. *et al.* Distribution, organization and expression of genes

concerned with anaerobic lactate utilization in human intestinal bacteria. *Microb Genom* **8** (2022).

15 Liu, J. *et al.* Remodeling of the gut microbiota and structural shifts in Preeclampsia patients in South China. *Eur J Clin Microbiol Infect Dis* **36**, 713-719 (2017).

16 Song, L. *et al.* Roseburia hominis Alleviates Neuroinflammation via Short-Chain Fatty Acids through Histone Deacetylase Inhibition. *Mol Nutr Food Res* **66**, e2200164 (2022).

17 Borren, N. Z. *et al.* Alterations in Fecal Microbiomes and Serum Metabolomes of Fatigued Patients With Quiescent Inflammatory Bowel Diseases. *Clin Gastroenterol Hepatol* **19**, 519-527.e515 (2021).

18 Ledwaba, S. E. *et al.* Enteropathogenic Escherichia coli Infection Induces Diarrhea, Intestinal Damage, Metabolic Alterations, and Increased Intestinal Permeability in a Murine Model. *Front Cell Infect Microbiol* **10**, 595266 (2020).

19 Gómez-Hurtado, I. *et al.* Role of interleukin 10 in norfloxacin prevention of luminal free endotoxin translocation in mice with cirrhosis. *J Hepatol* **61**, 799-808 (2014).

20 García-Lafuente, A., Antolín, M., Guarner, F., Crespo, E. & Malagelada, J. R. Modulation of colonic barrier function by the composition of the commensal flora in the rat. *Gut* **48**, 503-507 (2001).

21 Yang, Y. *et al.* Human Fecal Microbiota Transplantation Reduces the Susceptibility to Dextran Sulfate Sodium-Induced Germ-Free Mouse Colitis. *Front Immunol* **13**, 836542 (2022).

22 Kappler, K., Lasanajak, Y., Smith, D. F., Opitz, L. & Hennet, T. Increased Antibody Response to Fucosylated Oligosaccharides and Fucose-Carrying Bacteroides Species in Crohn's Disease. *Front Microbiol* **11**, 1553 (2020).

23 Henke, M. T. *et al.* Ruminococcus gnavus, a member of the human gut microbiome associated with Crohn's disease, produces an inflammatory polysaccharide. *Proc Natl Acad Sci U S A* **116**, 12672-12677 (2019).

24 Silverman, G. J., Deng, J. & Azzouz, D. F. Sex-dependent Lupus Blautia (Ruminococcus) gnavus strain induction of zonulin-mediated intestinal permeability and autoimmunity. *Front Immunol* **13**, 897971 (2022).

25 Gibson, D. L. *et al.* Maternal exposure to fish oil primes offspring to harbor intestinal pathobionts associated with altered immune cell balance. *Gut Microbes* **6**, 24-32 (2015).

26 Marriott, D., Stark, D. & Harkness, J. Veillonella parvula discitis and secondary bacteremia: a rare infection complicating endoscopy and colonoscopy? *J Clin Microbiol* **45**, 672-674 (2007).

27 Zhan, Z. *et al.* Overabundance of Veillonella parvula promotes intestinal inflammation by activating macrophages via LPS-TLR4 pathway. *Cell Death Discov* **8**, 251 (2022).

28 Gut microbiome of multiple sclerosis patients and paired household healthy controls reveal associations with disease risk and course. *Cell* **185**, 3467-3486.e3416 (2022).

Uncropped scans of all blots and gels in supplementary information

Supplementary Fig 6a: Genotyping of *Tlr4* $^{-/-}$ and *Tlr4* $^{+/+}$ mice.

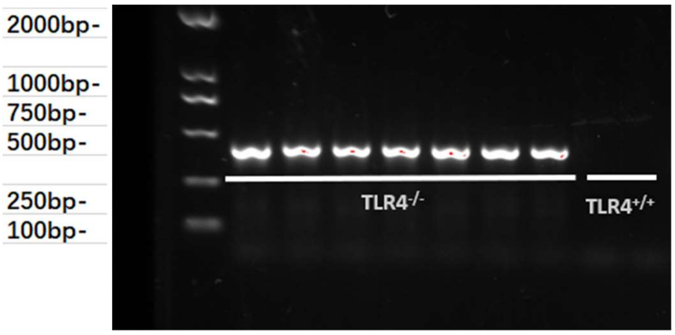

Supplementary Fig 6b: *Tlr4* knockdown efficiency validation.

Anti-TLR4

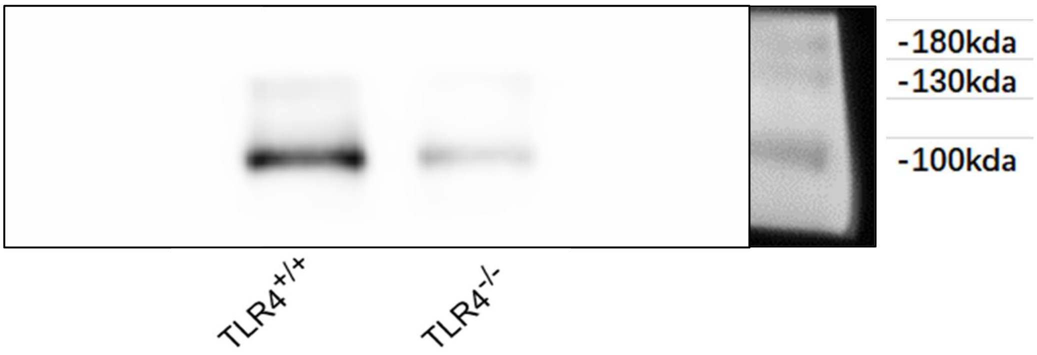

Anti-β-actin

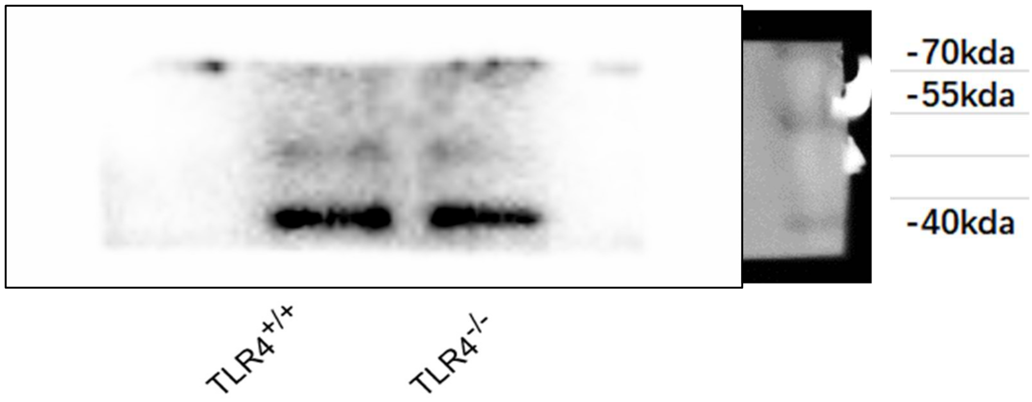

Supplement: Supplementary file 1 — Supplementary Information [file 41467_2023_44373_MOESM1_ESM.pdf]
