# Supplementary material for: Gut microbiota facilitate chronic spontaneous urticaria
Source: Nat Commun. 2024 Jan 2;15:112. doi: 10.1038/s41467-023-44373-x (PMC10762022; doi:10.1038/s41467-023-44373-x)
Supplement: Supplementary file 3 — Reporting Summary [file 41467_2023_44373_MOESM3_ESM.pdf]

nature portfolio

Corresponding author(s): Marcus Maurer, Jie Li.

Last updated by author(s): Nov 23, 2023

Reporting Summary

Nature Portfolio wishes to improve the reproducibility of the work that we publish. This form provides structure for consistency and transparency in reporting. For further information on Nature Portfolio policies, see our Editorial Policies and the Editorial Policy Checklist.

Statistics

For all statistical analyses, confirm that the following items are present in the figure legend, table legend, main text, or Methods section.

n/a	Confirmed	
☐	☒	The exact sample size (*n*) for each experimental group/condition, given as a discrete number and unit of measurement
☐	☒	A statement on whether measurements were taken from distinct samples or whether the same sample was measured repeatedly
☐	☒	The statistical test(s) used AND whether they are one- or two-sided<br>*Only common tests should be described solely by name; describe more complex techniques in the Methods section.*
☐	☒	A description of all covariates tested
☐	☒	A description of any assumptions or corrections, such as tests of normality and adjustment for multiple comparisons
☐	☒	A full description of the statistical parameters including central tendency (e.g. means) or other basic estimates (e.g. regression coefficient) AND variation (e.g. standard deviation) or associated estimates of uncertainty (e.g. confidence intervals)
☐	☒	For null hypothesis testing, the test statistic (e.g. *F*, *t*, *r*) with confidence intervals, effect sizes, degrees of freedom and *P* value noted<br>*Give P values as exact values whenever suitable.*
☒	☐	For Bayesian analysis, information on the choice of priors and Markov chain Monte Carlo settings
☒	☐	For hierarchical and complex designs, identification of the appropriate level for tests and full reporting of outcomes
☐	☒	Estimates of effect sizes (e.g. Cohen's *d*, Pearson's *r*), indicating how they were calculated

Our web collection on statistics for biologists contains articles on many of the points above.

Software and code

Policy information about availability of computer code

Data collection	Sequencing data was collected by Illumina NovaSeq 6000 system, Illumina Hiseq 2500,QTRAP 6500 mass spectrometer, Waters UPLC system, Agilent 8890B gas chromatography coupled to an Agilent 5977B/7000D mass selective detector,
Data analysis	Software used for data analysis included R platform(version 4.0.3), Masshunter(version 10.0.707.0, Agilent, USA), MultiQuant 2.0 software(SCIEX, Canada), Graphpad Prism(version 9.4.0, USA), Majorbio cloud platform(cloud.majorbio.com), Uparse software, qiime software(version 1.7.0), LEfSe software(version 1.0).

For manuscripts utilizing custom algorithms or software that are central to the research but not yet described in published literature, software must be made available to editors and reviewers. We strongly encourage code deposition in a community repository (e.g. GitHub). See the Nature Portfolio guidelines for submitting code & software for further information.

Data

Policy information about availability of data

All manuscripts must include a data availability statement. This statement should provide the following information, where applicable:

- Accession codes, unique identifiers, or web links for publicly available datasets
- A description of any restrictions on data availability
- For clinical datasets or third party data, please ensure that the statement adheres to our policy

Data that support the findings of this study are available within the paper and its Supplementary Information.The metagenomic sequencing files of human fecal

samples and the 16S rRNA gene sequencing files of fecal samples from experimental mice generated in this study have been deposited in the National Omics Data Encyclopedia (NODE). The metagenomic sequencing files of human fecal samples are available under project OEP002960 (http://www.biosino.org/node/review/detail/OEV000435?code=K4MN2WY7), the 16S rRNA gene sequencing files of fecal samples from experimental mice before and after antibiotic treatment were under project OEP002997 (http://www.biosino.org/node/review/detail/OEV000436?code=TN2WICKS).The mass spectrometry data generated in this study have been deposited in the China Nucleic Acid RepositoRy Database (CNGBdb, https://db.cngb.org/cnjb/) under accession CNP0005021."The experimental data generated in this study are provided in the Source Data file. Source data are provided with this paper

Research involving human participants, their data, or biological material

Policy information about studies with human participants or human data. See also policy information about sex, gender (identity/presentation), and sexual orientation and race, ethnicity and racism.

Reporting on sex and gender	Our study does not specifically apply to a particular sex or gender. We included the sex of patients or healthy controls in the study, and gender was not included in our study classification. In the design of this study, we matched the age and sex of CSU patients and healthy controls before sequencing to reduce confounding factors. Sex has been consistent with gender information determined by self-reporting appearance and identity card.
Reporting on race, ethnicity, or other socially relevant groupings	The included patients and healthy controls were from Hunan Province, China, and their economic status, educational status were collected based on the information provided by themselves and matched to minimize confounders.
Population characteristics	A total of 39 patients with CSU were included with a mean age of 35.87, mean BMI of 23.32kg/m2, female/male was 17/22. And 38 healthy controls were included with a mean age of 37.53, mean BMI of 23.18kg/m2, female/male was 16/22. Other information can be found in Supplementary Table 1.
Recruitment	Patients with CSU were recruited from the department of dermatology of Xiangya Hospital, Central South University, between Oct 2018 to Oct 2019. The diagnosis of CSU was carried out according toT he inclusion criteria for patients with CSU were: 1) Aged 18 to 65; 2) No oral or topical antihistamines within one month before sample collection; 3) No antibiotics, prebiotics, probiotics, glucocorticoids, omalizumab and other drugs within three months before sample collection; 4) No consumption of yogurt, pickles and other fermented foods within three days before sample collection; 6) Living in Changsha city for more than 1 year before sample collection. The exclusion criteria were: 1) Suffering from other subtypes of urticaria, such as symptomatic dermographism and acute urticaria, or allergic diseases; 2) With comorbid autoimmune disease (such as systemic lupus erythematosus, Sjogren's syndrome, thyroid problems, diabetes) and/or gastrointestinal symptoms; 3) Failure to collect fecal samples as required; 4) Lactation or pregnancy. the international guideline for urticaria. To match those patients above, the same criteria were applied for the recruitment of age- an sex-matched healthy controls (HCs) from the physical examination center of Xiangya Hospital of Central South University. Since the patients came from the dermatology outpatient department of the hospital, there may exist selection bias. We conducted random sampling to ensure that the samples were representative and could reflect the overall situation as much as possible.
Ethics oversight	The study was approved by the Ethics Committee of Xiangya Hospital of Central South University, Changsha, Hunan, China.

Note that full information on the approval of the study protocol must also be provided in the manuscript.

Field-specific reporting

Please select the one below that is the best fit for your research. If you are not sure, read the appropriate sections before making your selection.

☒ Life sciences ☐ Behavioural & social sciences ☐ Ecological, evolutionary & environmental sciences

For a reference copy of the document with all sections, see nature.com/documents/nr-reporting-summary-flat.pdf

Life sciences study design

All studies must disclose on these points even when the disclosure is negative.

Sample size	Our sample size selection was not determined by statistical methods, but based on previous study that found significant results with similar or even smaller sample sizes(Akram R et al. Int Immunopharmacol. 2018 Jun:59:168-173. doi: 10.1016/j.intimp.2018.04.007).
Data exclusions	Outliers in the measured data column were excluded.
Replication	In our metagenomic sequencing, 16s sequencing and targeted metabolomics, sequencing samples have enough numbers to ensure the reliability of the result analysis. In addition, all experiments, including animal experiments, cell experiments and molecular experiments, were repeated for three or more times using biologically independent samples, and all repeated experiments obtained similar results successfully, representative results were represented in our study.
Randomization	For clinical samples, assignment to experimental outcomes was randomized in the absence of significant differences in full covariates (age and sex). For animal experiments, mice of uniform age and sex were taken for all animal experiments and samples were randomly assigned.
Blinding	For all experiments using clinical samples, we ensured blinded outcome assessment. Disease diagnosis and inclusion/exclusion of patients were perfermed by two or more independent urticaria specialists at blinding status. The investigators were blinded to group allocation during data collection and/or analysis. . In animal experiments, we randomly group all the animals. For all samples and processing factors, we use a

uniform code to label them to avoid bias in judgment due to subjective reasons.

Reporting for specific materials, systems and methods

We require information from authors about some types of materials, experimental systems and methods used in many studies. Here, indicate whether each material, system or method listed is relevant to your study. If you are not sure if a list item applies to your research, read the appropriate section before selecting a response.

Materials & experimental systems

n/a	Involved in the study	
☐	☒	Antibodies
☐	☒	Eukaryotic cell lines
☒	☐	Palaeontology and archaeology
☐	☒	Animals and other organisms
☐	☒	Clinical data
☒	☐	Dual use research of concern
☒	☐	Plants

Methods

n/a	Involved in the study	
☒	☐	ChIP-seq
☐	☒	Flow cytometry
☒	☐	MRI-based neuroimaging

Antibodies

Antibodies used

(1) Mouse anti-dinitrophenyl (DNP) IgE (Sigma Chemical Co. (Missouri, USA); Cat#: D8406;Clone SPE-7);
(2) Anti-mouse CD117 (Kit)-APC, (BioLegend; Cat#: 161505; Clone: S18020A);
(3) Anti-mouse FcεRIα-PE/cy7, (BioLegend; Cat#: 334620; Clone: AER-37 (CRA-1));
(4) Anti-mouse CD45-APC/cy7, (BioLegend; Cat#: 103116; Clone: 30-F11);
(5) TLR4 mouse antibody, (Santa Cruz; Cat#: sc-293072; Clone: 25);
(6) Actin mouse antibody, (Santa Cruz; Cat#: sc-8432; Clone: C-2);
(7) MUC2 Rabbit mAb, (Abclonal; Cat#: A4767; Clone: ARC1012);
(8) ZO-1 Rabbit pAb, (Abclonal; Cat#: A0659; lot:5500020603);
(9) HRP Goat Anti-Mouse IgG (H+L), (Abclonal; Cat#: AS003;lot: 9300003001).
(10) Universal two-step Test Kit (Mouse/Rabbit Enhanced Polymer test system), (ZSGB-BIO, CN, Cat#: PV-9000, lot: 2203D1101)(For IHC).

Validation

Only antibodies validated for specificity by the manufacturers were used.
Antibodies Catalog validation statement:
(1) Mouse anti-dinitrophenyl (DNP) IgE (Sigma Chemical Co. (Missouri, USA); Cat#: D8406;Clone SPE-7). As per manufacturer website, 'Monoclonal Anti-Dinitrophenyl, clone SPE-7 has been used to detect the degranulation of rat mast cells by enzymolysis and fluorescence spectrophotometer'. Ref: ACS chemical biology, 10(2), 539-546 (2014-10-25);
(2) Anti-mouse CD117 (Kit)-APC, (BioLegend; Cat#: 161505; Clone: S18020A). As per manufacturer website, 'S18020A has been tested to block the ligand binding of SCF on c-Kit which is determined by the phosphorylation of ERK1/2 in c-Kit signaling';
(3) Anti-mouse FcεRIα-PE/cy7, (BioLegend; Cat#: 334620; Clone: AER-37 (CRA-1)). As per manufacturer website, 'Clone AER-37 (CRA-1) has been reported to bind the receptor even in the presence of IgE'. Ref: Kerr SC, et al. 2020. Clin Exp Allergy. 50:904;
(4) Anti-mouse CD45-APC/cy7, (BioLegend; Cat#: 103116; Clone: 30-F11). As per manufacturer website, 'Clone 30-F11 reacts with all isoforms and both CD45.1 and CD45.2 alloantigens of CD45'. Ref: 2022. Radtke AJ, et al. 2022. Nat Protoc. 17:378-401; Liu F, et al. 2012. Blood. 119:3295;
(5) TLR4 mouse antibody, (Santa Cruz; Cat#: sc-293072; Clone: 25). As per manufacturer website, near-infrared western blot analysis of TLR4 expression in Jurkat, HEL 92.1.7 and THP-1 whole cell lysates were detected using sc-293072. Ref: Hernandez, JC. et al. 2023. iScience. 26: 106254;
(6) Actin mouse antibody, (Santa Cruz; Cat#: sc-8432; Clone: C-2). As per manufacturer website, western blot analysis of Actin expression in C32, A-431, Sol8, BC3H1 and RAW 264.7 whole cell lysates were detected using sc-8432. Ref: Wang, X. et al. 2023. Pharmacol Res. 196: 106874;
(7) MUC2 Rabbit mAb, (Abclonal; Cat#: A4767; Clone: ARC1012). As per manufacturer website, A4767 was verified in HeLa, SGC-7901, Mouse stomach, and Rat large intestine. Ref: Ni YH et al. Chemosphere. 2021 Nov:282:130952.
(8) ZO-1 Rabbit pAb, (Abclonal; Cat#: A0659; lot:5500020603). As per manufacturer website, A0659 was verified in Rat lung. Ref: EMBO J. 2023 Jun 1;42(11):e112953;
(9) HRP Goat Anti-Mouse IgG (H+L), (Abclonal; Cat#: AS003; lot: 9300003001); As per manufacturer website, AS003 was verified in Mus musculus et al. Ref: Takahiro S et al. Nature. 2022 Aug;608(7922):421-428.
(10) Universal two-step Test Kit (Mouse/Rabbit Enhanced Polymer test system), (ZSGB-BIO, CN, Cat#: PV-9000, lot: 2203D1101). Ref: Merz H, et al. Lab Invest 1995;73:149-56.

Eukaryotic cell lines

Policy information about cell lines and Sex and Gender in Research

Cell line source(s)

Bone marrow derived mast cells (BMMCs) were derived from female C57 mice aged 4-6 weeks. RBL-2H3 was purchased from ATCC(CRL-2256).

Authentication	BMMCs determined by the Cytek Dxp Athena flow cytometer expressing both CD117 and FcεRI; The marker expression of RBL-2H3 were routinely checked and the efficiency of degranulation and histamine release were detected to identify the cell line.
Mycoplasma contamination	All cell lines tested negative for mycoplasma contamination.
Commonly misidentified lines (See ICLAC register)	No commonly misdentified cell lines were used.

Animals and other research organisms

Policy information about studies involving animals; ARRIVE guidelines recommended for reporting animal research, and Sex and Gender in Research

Laboratory animals	TLR4 -/- , TLR4 +/+ , BALB/c and C57BL/6 mice with specific pathogen free (SPF), aged 6-8 weeks or 4-6 weeks and weighing about 18-23g. BALB/c and C57BL/6 mice were purchased from Hunan SJA Laboratory Animal Co. Ltd. TLR4 -/- , TLR4 +/+ mice were donated from Ben Lv from the Key Laboratory of Sepsis Translational Medicine of Hunan and Department of Critical Care Medicine and Hematology of the 3rd Xiangya Hospital, Central South University. All animal experiments were conducted in the Department of experimental zoology of Central South University, a specific pathogen-free facility. The feeding environment of mice is: temperature 21-23°C, relative humidity 50% - 70%, 12h light / 12h dark alternating environment and had free access to standard feed and sterilized water. There are no more than 5 mice in each cage. After 1 week of habituation to the laboratory environment, mice were subjected to animal experiments.
Wild animals	No wild animals were involved.
Reporting on sex	Female animals were used for experiments.
Field-collected samples	This study did not involve samples collected from the field.
Ethics oversight	All experiments involved animals were approved by the ethics committee of experimental animal welfare of Central South University, with(approval No. 2021sydw0124).

Note that full information on the approval of the study protocol must also be provided in the manuscript.

Clinical data

Policy information about clinical studies

All manuscripts should comply with the ICMJE guidelines for publication of clinical research and a completed CONSORT checklist must be included with all submissions.

Clinical trial registration	No applicable.
Study protocol	No applicable.
Data collection	No applicable.
Outcomes	No applicable.

Flow Cytometry

Plots

Confirm that:

☒ The axis labels state the marker and fluorochrome used (e.g. CD4-FITC).

☒ The axis scales are clearly visible. Include numbers along axes only for bottom left plot of group (a 'group' is an analysis of identical markers).

☒ All plots are contour plots with outliers or pseudocolor plots.

☒ A numerical value for number of cells or percentage (with statistics) is provided.

Methodology

Sample preparation	Bone marrow derived murine mast cell(BMMCs) are derived from female C57 mice aged 4-6 weeks and cultured with the addition of murine IL-3 and murine SCF; dissociation of mouse skin into single cell suspensions by collagenase 4 digestion and washed with pbs.
Instrument	BD FACS LSR Fortessa Flow Cytometer.
Software	Flowjo software(V10.4).

Cell population abundance	BMMCs:The first gate contained >90% single cells, second gate contained >90% live cells, and third gate contained >90% CD117+ , FcεRIα+ cells. Mouse skin cell: The initial gate contained >90% single cells, second gate contained >50% live cells, third gate contained ~7% CD45+ cells, fourth gate contained 0.5%~7.5% CD117+, FcεRIα+ cells.
Gating strategy	FSC and FSCW-based fractionation of individual cells in the first gate, live cells were circled in the second gate by FSC and BV510+, CD45+ cells were circled in the third gate by APC/cy7 and SSC, CD117+/FcεRIα+ cells were circled through APC+ and PE/cy7+ in the fourth gate.

☒ Tick this box to confirm that a figure exemplifying the gating strategy is provided in the Supplementary Information.
